# Hybrid solar cell on a carbon fiber

**DOI:** 10.1186/s11671-016-1469-7

**Published:** 2016-05-23

**Authors:** Dmytro A. Grynko, Alexander N. Fedoryak, Petro S. Smertenko, Oleg P. Dimitriev, Nikolay A. Ogurtsov, Alexander A. Pud

**Affiliations:** V. Lashkaryov Institute of Semiconductor Physics, NAS of Ukraine, pr. Nauki 45, Kyiv, 03028 Ukraine; Institute of Bioorganic Chemistry and Petrochemistry, NAS of Ukraine, 50 Kharkivske shose, Kyiv, 02160 Ukraine

**Keywords:** Carbon fiber, CdS nanowire, Flexible nanobrush, Hybrid solar cells

## Abstract

In this work, a method to assemble nanoscale hybrid solar cells in the form of a brush of radially oriented CdS nanowire crystals around a single carbon fiber is demonstrated for the first time. A solar cell was assembled on a carbon fiber with a diameter of ~5–10 μm which served as a core electrode; inorganic CdS nanowire crystals and organic dye or polymer layers were successively deposited on the carbon fiber as active components resulting in a core-shell photovoltaic structure. Polymer, dye-sensitized, and inverted solar cells have been prepared and compared with their analogues made on the flat indium-tin oxide electrode.

## Background

**Fig. 1:**
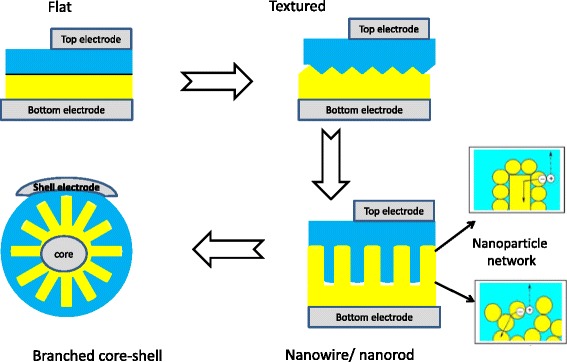
Illustration of evolution of a flat heterojunction (HJ) for charge separation into various HJ configurations leading to the increased interface area: textured, nanorod/nanowire/nanoparticle network, branched core-shell morphology

Hybrid organic-inorganic solar cells based on organic molecules and inorganic semiconductor crystals, which serve as electron donor and electron acceptor, respectively, attract great attention due to the mutual advantages of the both materials used in the same device [1]. One of the key factors influencing the effective work of the hybrid photovoltaic devices is the interface area between the counterparts. The higher is the interface area, the higher amount of excitons dissociates into free carriers per the same amount of the absorbed photons. Therefore, the design of interface geometry is of crucial importance for charge carrier generation and collection. The evolution of typically applied geometries/morphologies can be illustrated by the scheme in Fig. 1.

The advantages of the nanowire (NW) or nanorod morphology compared to the textured or flat one have been proved in our previous works [2–5]. In particular, application of semiconductor NW arrays in solar cells leads to better light absorption due to the reduced reflection and stronger light trapping (so-called shadow effect) and improvement of charge collection from the active layer, since charge carriers move straight to the respective electrode through a NW crystal [6].

Among typically used inorganic NW components of the hybrid PV cells, CdS nanocrystals and their aligned arrays have attracted much attention due to their relative cheapness, comparatively easy preparation, and adhesion to different surfaces as well [7, 8]. Over the past few years, tremendous efforts have been made to decrease the size and to control the shape of CdS nanocrystals, and a number of new methods were reported for the synthesis of CdS nanocrystals integrated into low-dimensional nano- and microstructures [9, 10]. Specifically, a significant extension of surface area of a NW CdS array can be obtained by using the branched morphology. This approach has resulted in effective application of branched and hyper-branched semiconductor nanocrystals in energy conversion devices [11], sensors [12], electronic logic gates [13], etc. The advantage of the branched morphologies stems from an increased surface of such nanocrystals and therefore from higher contribution of processes at the interface of the nanocrystal and the environment. Therefore, the modern trends in photovoltaics reveal the gradual changes from the flat morphology of hybrid heterojunctions to the textured one and to the branched NW configuration (Fig. 1).

Although the solar cells based on CdS nanocrystals usually do not demonstrate superior power conversion efficiency (PCE) (Table 1), this material is convenient to study as a proof of concept for new cell geometries and to model hybrid interface properties [4, 14–16] due to the abovementioned advantages.

**Table 1:** Overview of some recent results in the world for PV cells based on CdS.

Composition of hybrid heterojunction	Morphology of heterojunction (HJ)	PCE, %	Illumination level	Reference
CdS/PPY	Nanorod core—polymer shell HJ	0.018	6.05 mW/cm^2^	Y.Guo et al., J. Phys. Chem. Lett.1 (2010) 327
CdS/P3HT	Polycrystalline film—polymerHJ	0.15	AM1.5	S.A. Yuksel et al., Thin Solid Films 540 (2013) 242.
CdS/P3HT	Nanocrystal—polymer BHJ	4.1	AM1.5	S.Ren et al., Nano Letters, 11 (2011) 3998
CdS/MEH-PPV	Nanocrystal—polymer BHJ	0.6	AM1.5	Y.Kang, D.Kim, Sol. Energ. Mat. Sol. Cells 90 (2006) 166
CdS/MEH-PPV	NW array—polymer BHJ	0.035	AM1.5	X.Jiang et al., Sol. Energ. Mat. Sol. Cells 94 (2010) 338.
CdS/MEH-PPV	NW array—polymer BHJ	1.17	AM1.5	L. Wang et al., J. Phys. Chem. C 111(2007) 9538
CdS/MEH-PPV	NW array—polymer BHJ	1.62	AM1.5	J.-C. Lee et al., Electrochem. Commun. 11 (2009) 231.
CdS/N719/P3HT	Dye-sensitized polycrystalline film	1.31	AM1.5	M. Zhong et al., Sol. Energ. Mat. Sol. Cells 96 (2012) 160.
CdS/Rhodamine B	NW array loaded with dye in DSSC	0.12 × 10^−5^	AM1.5	B. Sankapal et al., J. Alloy. Compd. 651 (2015) 399.
CdS/Cu_2_S	Nanorod core-shell HJ	5.4	AM1.5	J.Tang et al., Nature Nanotechnol. 6 (2011) 568.
CdS/CdTe	Nanorod core-shell HJ	6.0	AM1.5	Z. Fang et al., Nature Mater. 8 (2009) 648
CdS/Cu(In,Ga)Se_2_	Nanorod core-shell HJ	6.18	AM1.5	W.-C. Kwak et al., Cryst. Growth Des. 10 (2010) 5297.

In this work, we discuss the original method of design of nanoscale hybrid solar cells of four different types based on a single carbon fiber (CF) as a core electrode supporting active layers of the developed solar cell in the form of subsequent shells. It should be noted that fiber-shaped solar cells have attracted great attention recently in view of their potential integration into large-scale and low-cost textile and wearable electronic devices. It has been shown that carbon-based materials can be used both as a core electrode [17, 18] and as a counter-electrode [19–22] in respective solar cells. In this work, we use a novel approach to construct a shell in the form of a brush of radially oriented CdS NWs around a single CF. In such a cell, the flexible hybrid core-shell CF-CdS nanobrush serves as the inorganic acceptor component, whereas organic shell of Zn phthalocyanine (ZnPc) or poly(3-hexylthiophene) (P3HT) was used as a donor light-absorbing overlayer.

## Methods

### Materials

Single carbon fibers (CFs, diameter was about 5–10 μm and the length 15–30 mm) were taken out of the commercial carbon cloth LU-3 (Ukraine) produced by carbonization of polyacrylonitrile cloth and characterized with Young’s modulus E~250 GPa and tensile strength 2.5–3.0 GPa. The CF separation was handled using an optical microscope with the help of a homemade microinstrument. Resistivity of CF was *ca*. 3.8 · 10^−3^ Ohm · cm. Studies on fiber flexibility showed that a single CF could be bent at 180° without fiber cracking when the bent radius is as small as 500 μm. CdS powder of the chemical grade (purity 99.998 %) was used for the growth of NW crystals on the surface of a single CF. Zinc 2,9,16,23-tetra-tert-butyl-29H, 31H-phthalocyanine (ZnPc-4R) (Sigma-Aldrich) or P3HT (Rieke Metals) served as organic donor counterparts. To prepare the top electrode poly(3,4-ethylenedioxythiophene)-poly(styrenesulfonate) (PEDOT:PSS) (Aldrich) has been drop-cast from the 1.3 wt.% water dispersion.

### Preparation of the CdS/CF Nanobrush Structure

**Fig. 2:**
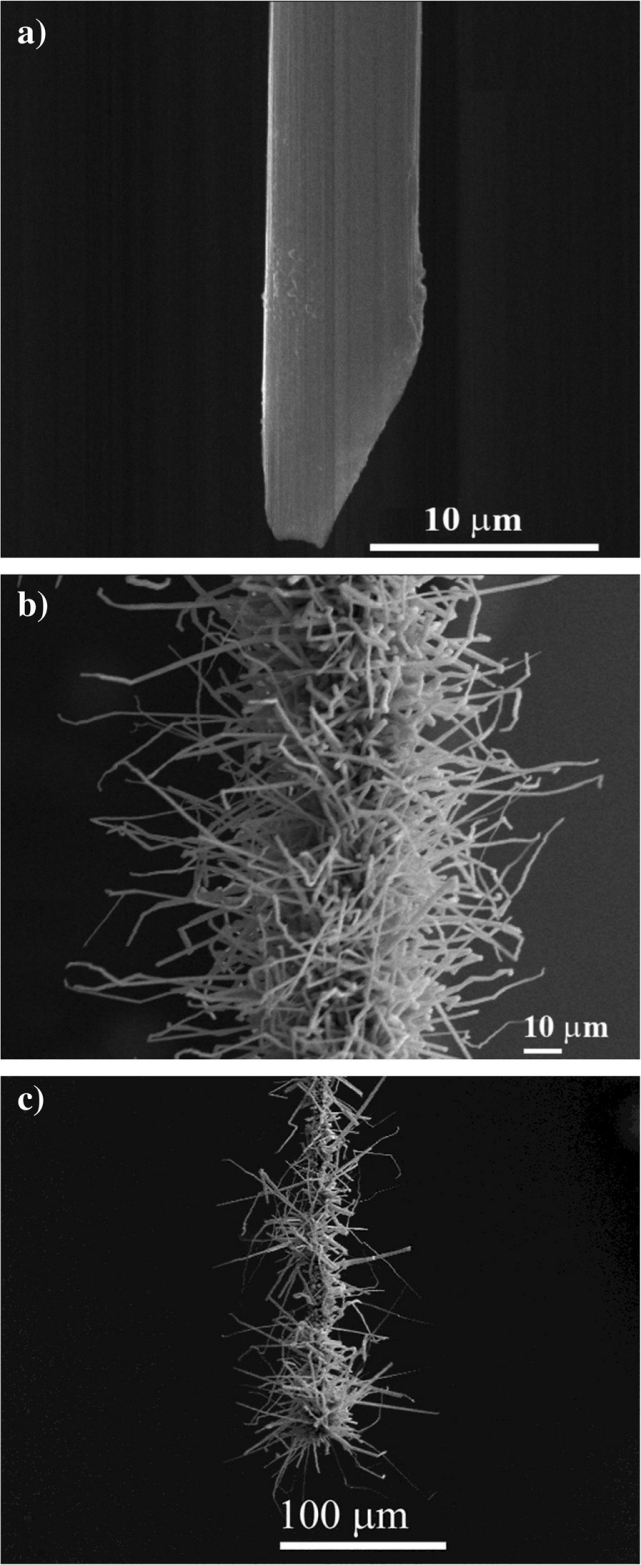
SEM images of **a** bare CF and **b**, **c** CdS NW array on a single CF under different magnifications

Details of the growth of the CdS NW array on CF are given elsewhere [23]. In short, the synthesis was performed by vapor-solid (VS) condensation technique [10] in a high-temperature reactor inside the vacuum chamber, at temperatures between 650 and 750 °C with the basic pressure in the chamber of ~10^−5^Torr. No gold or other metal islands were used as nucleation centers/seeds for the nanocrystal growth, and CdS nanocrystals were grown directly on the bare CF surface (Fig. 2). The adhesion of CdS crystals to CF was checked by electrical measurements which showed an ohmic or quasi-ohmic character of the contact between CdS and CF [17].

### Solid-State Dye-Sensitized Solar Cell (SSDSSC)

A layer of ZnPc-4R in 1 wt% ethanol was drop cast onto the CdS nanobrush surface to prepare the core-shell CF/CdS/ZnPc-4R active heterojunction layer followed by deposition of the PEDOT:PSS top electrode for the SSDSSC formation. Indium pads with corresponding leads were clamped directly to a metallic holder attached to CF and to PEDOT:PSS as counter-electrode, respectively, for the electrical measurements.

### Electrochemical Dye-Sensitized Solar Cell (DSSC)

A drop of Na_2_S-S electrolyte (10 μl) was drop-cast onto the same CF/CdS/ZnPc-4R nanobrush structure and covered by glassy carbon electrode via 15 μm PTFE spacer.

### Solid-State Polymer-Sensitized Solar Cell (SSPSSC)

P3HT from 1 wt% chlorobenzene solution was drop-cast on the CF/CdS nanobrush array followed by annealing during 10 min (110 °C) under argon atmosphere. It should be noted that the polymer deposition can somewhat affect the fragile CdS NWs (Fig. 2). The analogous PV cells have been prepared on the flat indium-tin-oxide (ITO) surface by a similar successive deposition of CdS NW array, organic overlayer, and PEDOT:PSS electrode to compare PV performance of the cells of the different geometry and similar CdS NW arrays.

### Inverted Solar Cell (ISC)

P3HT and fullerene derivative [6, 6]-phenyl-C61-butyric acid methyl ester (PCBM) mixture (1:2 molar ratio) from 1 wt% chlorobenzene solution was drop-cast on the CF/CdS nanobrush array followed by annealing during 10 min (110 °C) under argon atmosphere. The analogous PV cells have been prepared on the flat ITO surface by a similar successive deposition of CdS NW array, organic overlayer, and PEDOT:PSS electrode to compare PV performance of the cells of the different geometry and similar CdS NW arrays.

### Characterization

*I-V* measurements were performed by using a HP 4140B source meter device interfaced to a computer. White light illumination of the samples was provided by a 50-W halogen lamp. The results below are referred to light intensity of 100 mW/cm^2^ if not indicated otherwise. The *I-V* curves were processed by differential approach to analyze their fine structure [24–26]. This approach allowed us (i) to determine the differential slope *α* according to the equation

1 $$ \alpha (V)=\frac{d(lgI)}{d(lgV)}=\frac{dI}{dV}\times \frac{V}{I}; $$

(ii) to describe the *I-V* curves by the dependence

2 $$ I(V)\sim {V}^{\alpha } $$

(iii) to determine the differential slope of the second order γ according to the equation

3 $$ \gamma (V)=\frac{d\left( \lg \alpha \right)}{d\left( \lg V\right)}=\frac{d\alpha }{dV}\times \frac{V}{\alpha }; $$

(iv) to describe the *I-V* curves by the dependence

4 $$ I(V)\sim \exp \left({V}^{\gamma}\right); $$

(v) to determine the ideality factor η by substitution of current density from the diode equation

5 $$ j(V)={j}_{\mathrm{S}}\left( \exp \left\{\frac{eV}{\eta kT}\right\}-1\right)\cong {j}_{\mathrm{S}} \exp \left\{\frac{eV}{\eta kT}\right\} $$

to Eq. (1), which results in

6 $$ \eta =\frac{e}{kT}\cdot \frac{V}{\alpha }; $$

where *k* is the Boltzmann constant, *e* the electron charge, and *T* the absolute temperature.

The major charge carrier type in CF was determined by the hot-probe experiment. In this method, a bundle of CFs was fixed by two crocodile clamps and then heated from the one side while measuring the potential difference between the clamps. Depending on the sign of the potential difference, the main type of charge carriers was determined.

Morphologies of the samples were studied by scanning electron microscopy (SEM) using JEOL JSM35, JXA-8200 instruments and by optical microscope ULAB XY-B2.

## Results and Discussion

The needle-like crystals of CdS were obtained at the surface of the CF (Fig. 2). Their growth mechanism was discussed in detail elsewhere [10]. Particularly, it was shown that the formation of CdS NWs on the CF proceeds through vapor-solid (VS) mechanism due to adsorption of the reactive gas phase directly on dangling bonds, polar groups, defects, etc., along the CF surface. Based on this approach, a rather robust contact of the CdS nanocrystals to CF can be formed, resulting in a quasi-ohmic behavior of the CF/CdS NW heterostructure. From the SEM images of the heterostructures made (Fig. 2), it was estimated an average diameter and length of the needle-like CdS crystals to be 300–700 nm and up to 10 μm, respectively; in some cases, the crystal length extended up to 50 μm. The estimated surface density was several CdS NW crystals per square micron.

**Fig. 3:**
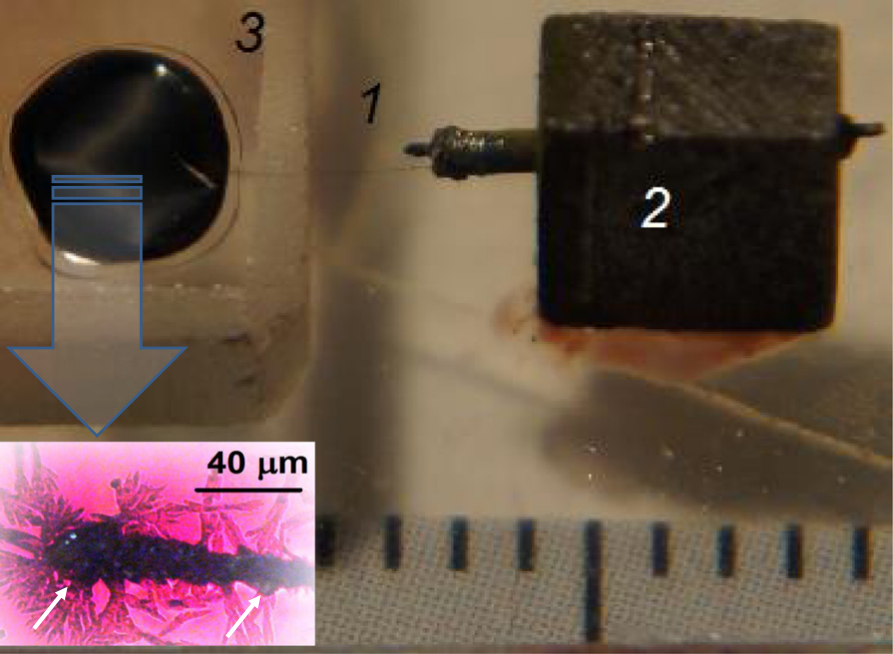
The assembled PV device: *1* CF, *2* supporting conductive block, *3* ITO/PEDOT:PSS counter-electrode. The *insert* shows a part of the CF/CdS NW assembly after soaking with the polymer solution; some NWs become broken and the *arrows* show CF thickening due to the polymer adsorption

**Fig. 4:**
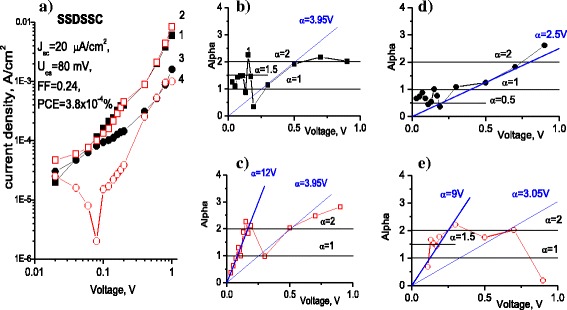
**a** *I-V* characteristics and **b**–**e** *α*(V) dependence of SSDSSC (CF/CdS/ZnPc-4R/PEDOT:PSS): *squares* correspond to positive and *circles* to negative potential on CF, respectively; *black curves* correspond to the dark and *red* ones to illumination conditions, respectively

**Fig. 5:**
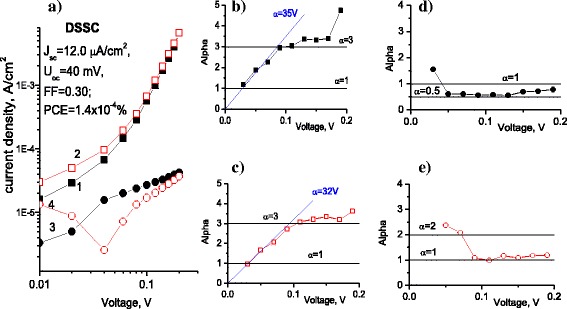
**a** *I-V* characteristics and **b**–**e** *α*(V) dependence of DSSC (CF/CdS/ZnPc-4R/glassy carbon): *squares* correspond to positive and *circles* to negative potential on CF, respectively; *black curves* correspond to the dark and *red* ones to illumination conditions, respectively

The prepared CF/CdS nanobrush structure served as an electron acceptor component of the PV cell and was then covered by an additional layer of donor organic material (ZnPc-4R, P3HT or P3HT:PCBM) resulting in the penetration of the donor material into the porous CdS structure and formation of bulk heterojunction (BHJ) micron-sized solar cells (Fig. 3). The prepared different types of the hybrid CF/CdS nanobrush cells have somewhat different principles of operation, particularly, the different role of CdS layer in each type of the above cells, and therefore different PV performances can be expected. In SSPSSC and SSDSSC, CdS acts as an electron acceptor, while it plays the role of an electron-selective (hole-blocking) layer to direct electrons from the organic counterpart to cathode in DSSC and ISC assemblies. In SSPSSC, SSDSSC, and DSSC, an exciton dissociates at the organic-inorganic CdS interface, while in ISC, an exciton dissociates at organic-organic interface followed by drift of electrons to the CdS layer. More details about operation of the PV cell of the same compositions but assembled on the flat supporting electrodes can be found elsewhere [4]. The PV performance was found to be also dependent both on the type of the contact (solid or liquid) of active BHJ structure with counter hole-collecting electrode (anode) and on the organic donor material as well. In particular, the SSDSSC (CF/CdS/ZnPc-4R/PEDOT:PSS) showed *V*_oc_ of ~0.08 V and *I*_sc_ of 20 μA/cm^2^ (Fig. 4), while the same charge-generating hybrid structure of CF/CdS/ZnPc-4R in the electrochemical DSSC led to somewhat worse PV characteristics with *V*_oc_ of 0.04 V and *I*_sc_ of ~12 μA/cm^2^ (Fig. 5). However, both photocurrent and open-circuit voltage in the SSDSSC degraded rapidly during the first minutes of measurements under illumination by factors of ~3 and ~2, respectively, most probably due to photooxidation processes, since CdS is known as a strong catalyst under certain conditions [27, 28]. The PV parameters of the above cells are given here when the transient processes have practically finished; then, these parameters for the SSDSSC were found to remain stable at least during 1 week. It should be noted that the photocurrent density and PCE, respectively, can be evaluated in the CF cells only approximately because of the developed surface of CdS/dye interface whose area could not be calculated exactly. The surface area through which the photocurrent was measured was roughly evaluated as a product of the CF diameter and the CF length covered by the PEDOT:PSS counter-electrode.

It should be noted that the SSDSSC based on the hyper-branched core-shell morphology allowed us to get an increased PCE by more than two orders of magnitude as compared with the cells prepared on the flat ITO electrode which had PCE less than 10^−5^ % (Table 2). We suggest that the improved PV performance of the core-shell SSDSSC is just due to the increased CdS-dye interface area because of the hyper-branched morphology which is more prominent when it grows up in the radial direction than on the flat surface and because insulating dye layer better prevents shortcutting problem in SSDSSC in contrast to the polymer-containing cells which will be discussed later. However, the performance of SSDSSC can be also overestimated due to possible underestimated surface area upon calculation of the photocurrent density as was discussed above. Nevertheless, we expect that the future optimization of the cell structure, i.e., application of the hole-transporting layer (HTL) and a better contact of the top electrode will further improve the PV performance of solar cells of this type.

**Table 2:** Comparison of PV performance of the different types of CdS NW array solar cells prepared on CF core and flat ITO electrode, respectively.

Type of solar cell	Organic material used	*V*_oc_, V CF core-shell/flat geometry	*I*_sc_, μA/cm^2^ CF core-shell/flat geometry	FF CF core-shell/flat geometry	PCE, % CF core-shell/ flat geometry
SSDSSC	ZnPc-4R	0.08/0.1	20/0.01	0.24/0.23	3.8 · 10^−4^/1.0 · 10^−6^
DSSC	ZnPc-4R	0.04/0.292	12/114	0.30/ 0.32	1.4 · 10^−4^/1.1 · 10^−2^
SSPSSC	P3HT	0.04/0.347	0.9/100	0.28/0.30	1.0 · 10^−5^/1.0 · 10^−2^
ISC	P3HT:PCBM	0.062/0.43	120/310	0.22/0.25	1.5 · 10^−2^/3.3 · 10^−2^

**Fig. 6:**
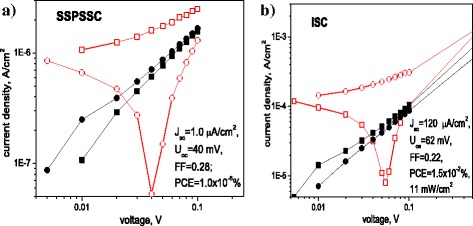
*I-V* characteristics of **a** SSPSSC (CF/CdS/P3HT/PEDOT:PSS) and **b** ISC (CF/CdS/P3HT:PCBM/PEDOT:PSS): *squares* correspond to positive and circles to negative potential on CF, respectively; *black curves* correspond to the dark and *red* ones to illumination conditions, respectively. Illumination was 11 mW/cm^2^ for **b**

PV performance of SSPSSC (CF/CdS/P3HT/PEDOT:PSS) was found to show poorer characteristics, with *V*_oc_ of 0.04 V and *I*_sc_ of about 1 μA/cm^2^ (Fig. 6a), which is far less than corresponding characteristics of the counterpart PV cell based on the flat ITO electrode (Table 2). At the same time, the ISC (CF/CdS/P3HT:PCBM/PEDOT:PSS, measured under the light intensity of 11 mW/cm^2^) showed *V*_oc_ of 0.062 V and *I*_sc_ of 120 μA/cm^2^ (Fig. 6b). Although such a performance is poorer as compared with the best PV cells based on P3HT:PCBM known in the literature, it is comparable with our flat counterpart PV cell of the same composition (Table 2). Therefore, the obvious advantage of the core-shell morphology in respect to the flat one was found only for the SSDSSC which showed superior characteristics as compared with the reference CdS NW cell prepared on the flat ITO electrode and showing *V*_oc_ of 0.08 V and *I*_sc_ of 20 μA/cm^2^, while fill factor of all developed cells was approximately the same, in the range of 0.22 to 0.30 (Table 2).

Analysis of *I-V* characteristics was performed for the related SSDSSC and DSSC structures (which have the same charge generating CF/CdS/ZnPc-4R heterostructure), which is illustrated in Figs. 4 and 5. In most cases, *I-V* curves can be described by power dependence behavior, excepting the currents under illumination in the range from 0 to 0.2 V, where the *I-V* curve can be described by exponent with *γ*(*V*) = 1 and *α*(*V*) = 12 V (Fig. 4c, Eqs. (3) and (5)).

Formally, we can describe the *I-V* curve also by exponent with *γ*(*V*) = 1 and *α* (*V*) = 3.95 V in the range between 0.2 and 0.5 V (Fig. 5b) with negative potential on CF and between 0.5 and 0.7 V with positive potential on CF under illumination and in the dark (Fig. 4c). The large part of *α* curves follows the power dependence with *α* = 2 (Fig. 4b, e) that corresponds to monomolecular recombination with *p >> n*, i.e., in the structure the concentration of injected charge carriers is not enough for bimolecular recombination with *p ≈ n* which corresponds to *α* = 1.5. The regime of bimolecular recombination is absolutely necessary for solar cells, where both types of charge carriers contribute equally to the photocurrent. Such a behavior can be seen in a very small range of voltages from 0.1 to 0.2 V (Fig. 4b, e). Here, the formal approximation by exponent with *α* = 3.95 (Fig. 4b) or even with *α* = 12 gives ideality factor (according to Eq. (6)) *η* = 9.77 and *η =* 3.21, respectively, that is far from ideal diode characteristic. So, for better PV performance of this type of structure, it would be necessary, first, to improve the injection of both types of charge carriers from the contacts and, second, to decrease bulk resistance that will improve the ideality factor of the structure.

In the case of the DSSC structure (Fig. 5), the current behavior with negative potential on CF can be described by exponential function in the range from 0 to 0.1 V. Here the *I-V* curve can be described by exponent with *γ*(*V*) = 1 and *α*(*V*) = 35 V in the dark and *α*(*V*) = 32 V under illumination. It gives the ideality factor (according to Eq. (3)) *η =* 1.10 and *η =* 1.2, respectively, which is quite close to the ideal diode *I-V* characteristics described by Eq. (2). Then, the current behavior follows power function with *α* = 3, that corresponds to high injection into the dielectric medium when concentration of injected charge carriers is much more than the bulk one [25, 29]. Thus, injection behavior in this case is better than in SSDSSC (Fig. 4). With positive potential on CF (Fig. 5d), there are saturation regions with *α* = 0.5. Therefore, in this case, there is almost an ideal diode behavior of *I-V* curves. In order to improve PV performance of this structure, therefore, a substantial decrease of bulk resistance is necessary.

The above analysis of the *I-V* curves suggests that in the both nanobrush structures (SSDSSC and DSSC), the interface of CdS NWs with ZnPc-4R plays the major role in generation of photoexcited charge carriers. This suggestion agrees well with the fact that at high voltages, the difference between dark and light currents is very small (Figs. 4 and 5). At the same time, the notable photosensitivity is observed only at small voltages (up to ~0.08 V). Therefore, despite better performance of the SSDSSC found in this study, the DSSC structure displays better PV behavior than the SSDSSC from the viewpoint of charge injection and therefore it could be more attractive for further improvement in PV application.

It should be noted that here we demonstrate only a proof of concept of new nanoscale solar cells using a commercial carbon textile (i.e., carbon cloth in our case). Naturally, the cells should be optimized because there are several factors which limit their performance. First, application of CF itself implies that its work function should be consistent with the energy levels of other materials of the solar cell assembly. Particularly, the difference in the work function of the anode and the cathode is the driving force (the built-in potential) in BHJ solar cells which moves the electrons and holes in the opposite directions. The exact work function of CF is not known but it should be close to the other carbon-related materials. It is known, for example, that the work function of carbon nanotubes is about 5.0 eV, while that of highly oriented pyrolytic graphite is 4.8–4.9 eV [30, 31]. Therefore, one can suggest that the CF material has work function around 5 eV as well. But this value is close to that of PEDOT:PSS (5.0 eV) which is the counter electrode in the above system. Therefore, there is a very small driving force which separates electrons and holes in the PV cell of that type, which can be the reason of the observed small open-circuit voltages, respectively. Moreover, the hot-probe experiment has revealed that the major charge carriers in the used CF are holes that can worsen collection of electrons from CdS and therefore, in general, leads to disadvantages in charge collection since the major charge carriers in the PEDOT:PSS counter electrode are holes as well. On the other hand, this situation suggests a possibility to solve the above problem and to improve the PV performance through replacement of CdS by p-type semiconductor, for example CdTe, along with the change of PEDOT:PSS counter electrode by that possessing a low work function and electrons as the major charge carriers.

The other drawback of the above system is the loosely distributed CdS array on the CF surface, possessing large pores, which allows for polymer to penetrate deeply into the CdS nanobrush structure (see insert in Fig. 3) and to contact with the CF electrode and thus to contribute to undesirable current leakage. This drawback is clearly seen upon comparison of performance of solar cells prepared on the CF and on the flat ITO electrode. The latter geometry provides tighter CdS layer and better solution of the shortcutting problem which is particularly important in cells where the polymer layer (SSPSSC and ISC) or a liquid contact (DSSC) is used. As a result, a significant increase in the open-circuit voltage can be achieved (Table 2). Therefore, deposition of a tighter CdS shell layer around the CF core electrode is necessary to solve the above problem. Finally, there is a problem with the reliable contact between the organic layer and the top PEDOT:PSS electrode. We have revealed that casting of the PEDOT:PSS electrode from a solution leads to shortcutting problem, but the mechanically pressed PEDOT:PSS film onto the top of the assembly does not provide a tight contact. The above contact problems affect reproducibility of the device substantially, with variation of photocurrent within one order of magnitude depending on the contact quality (cast or pressed, etc.).

We hope that the future solution of the above problems will result in the flexible fiber-based PV cell possessing a better performance.

## Conclusions

In this work, we have demonstrated the textile-based hybrid solar cells using inorganic CdS nanocrystals and organic dye or polymer as photoactive components. As a textile component, the conductive CF taken from the carbon cloth was used. We have showed that a single CF can serve as an aligned core electrode for the growth of CdS NW array followed by deposition of organic donor layer (ZnPc-4R, P3HT or P3HT:PCBM) resulting in active BHJ layers in new micron-sized core-shell PV structures.

It was found that behavior of charge carriers in the SSDSSC structure obeys mainly the power-law dependence with *α* = 2 that corresponds to the first-order (monomolecular) recombination with *p* >> *n*, that means that concentration of the injected minority charge carriers is not enough in the structure. In the case of the DSSC structure, the charge carriers behavior follows the cubic dependence with *α* = 3. In both structures based on hyperbranched CdS nanobrushes, the interface of CdS nanowires with ZnPc-4R plays the main role in formation of photo-generated charge carriers.

Analysis of the *I-V* curves allowed us to suggest the ways of optimization of the above PV structures, namely, to substantially decrease bulk resistance in SSDSSC and DSSC and to improve injection of both types of charge carriers from the contacts in case of SSDSSC. In SSPSSC and ISP, the use of the polymer layer requires a tighter CdS layer around the core CF electrode to escape shortcutting problems. The replacement of CdS for the p-type semiconductor would be useful as well for the future experiments.

Although a great work in order to get better performance of the respective PV cells should be undertaken, in principle, the developed technology can be considered as a major step towards “photovoltaics on curtains” [32].
